# Whole genome sequence data of an Antarctic bacterium, *Arthrobacter* sp. ES1 from the Schirmacher Oasis, East Antarctica

**DOI:** 10.1016/j.dib.2023.109052

**Published:** 2023-03-08

**Authors:** Chui Peng Teoh, Nur Athirah Yusof, Cahyo Budiman, Yoke Kqueen Cheah, Clemente Michael Vui Ling Wong

**Affiliations:** a Biotechnology Research Institute, Universiti Malaysia Sabah, 88400 Kota Kinabalu, Sabah, Malaysia; b Department of Biomedical Science, Faculty of Medicine and Health Sciences, Universiti Putra Malaysia, 43400 UPM Serdang, Selangor Darul Ehsan, Malaysia

**Keywords:** *Arthrobacter*, Antarctica, Whole genome sequencing, Genome

## Abstract

*Arthrobacter* is a coryneform bacterium in the family of *Micrococcaceae. Arthrobacter* species isolated from hostile environments are capable of producing interesting bioactive compounds, some of which may be a new class of antibiotics*.* Here, we present the complete genome sequence of *Arthrobacter* sp. ES1 isolated from Schirmacher Oasis in East Antarctica. Genomic DNA sequencing was performed using the Illumina MiSeq sequencer. *Arthrobacter* sp. ES1 has a genome size of 3,964,927 bp and a GC content of 65.73%. The raw genome sequences have been deposited in the NCBI Sequence Read Archive database under the accession number, SRR20664316.

**Specification Table**

**Table:** 

Subject	Biology

Specific subject area	Microbiology and genomics
Type of data	Table Figure
How the data were acquired	The genomic library was constructed using Nextera® XT DNA Sample preparation kit. Genome sequencing was performed using 300 cycles of Miseq® Regent Kit v2 and Illumina MiSeq Platforms. Raw sequencing data were trimmed and filtered using SolexaQA and bowtie2 tools [1,2]. *De novo* assembly was performed by using Velvet [3]. The genome completeness was assessed using BUSCO [4] tool. Genome annotation was performed using an online web tool, Rapid Annotation using Subsystem Technology (RAST) [5].
Data format	Raw Filtered Analyzed Assembled Annotated
Description of data collection	Genomic DNA *Arthrobacter* sp. ES1 was extracted using QIAGEN® DNeasy Blood and tissue kit.
Data source location	*Arthrobacter* sp. ES1 was isolated from a snow sample collected from the Schirmacher oasis (S70° 07’ 2.3” E 22۫° 55’ 46.5”), East Antarctica.
Data accessibility	The data is hosted at National Center for Biotechnology Information Bioproject: https://www.ncbi.nlm.nih.gov/bioproject/PRJNA344835 Biosample: https://www.ncbi.nlm.nih.gov/biosample/SAMN05712594 NCBI GenBank Accession Number: NZ_MQTO01000000 https://www.ncbi.nlm.nih.gov/nuccore/2119834887 Repository name: NCBI SRA database Data identification number: SRR20664316 Direct URL to data: https://trace.ncbi.nlm.nih.gov/Traces/sra/?run=SRR20664316

## Value of the Data

• Whole genome sequence data can be used to identify *Arthrobacter* sp. ES1 and determine whether it is a new species.
• Whole genome sequence data of strain ES1 can be useful for comparative genomic studies with other *Arthrobacter* species.
• Unravelling the genome of strain ES1 may aid in the discovery of novel bioactive compound-coding gene clusters.

## 1 Objective

*Arthrobacter* sp. Are ubiquity present in the environment, they are frequently isolated from the soil. *Arthrobacter* sp. strains have exceptional survival abilities. They have been isolated from a variety of harsh environments, including radioactive and chemically contaminated sites as well as the polar regions. These *Arthrobacter* sp. are capable of metabolizing and resisting environmental hazards and heavy metals [6]. In addition, polar environments have been proposed as a source of novel bioactive compounds. Sixteen bacterial strains that produce antibiotics have been isolated from the central Arctic Ocean by Wietz et al., [7]. Seven of these *Arthrobacter* spp. can produce arthrobacilins, A and C under different growth conditions [7]. Our objective was to sequence, assemble, and annotate the genome of *Arthrobacter* sp. ES1, which would allow us to discover new bioactive compounds and conduct evolutionary research.

## 2 Data Description

**Fig. 1:**
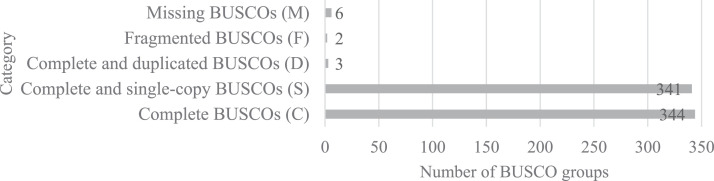
Quality for the draft genome of *Arthrobacter* sp. ES1 was assessed by using the BUSCO tool tested with actinobacteria_odb9 lineage.

**Fig. 2:**
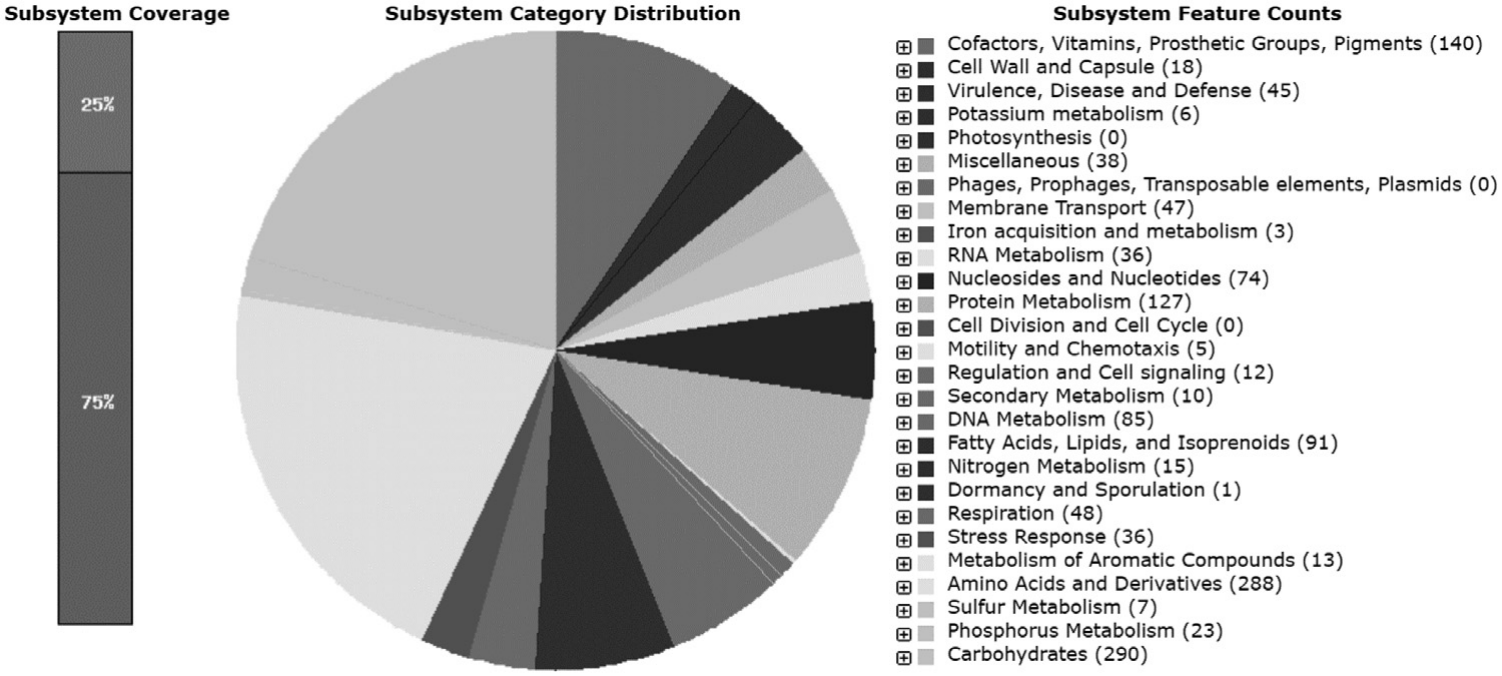
Subsystem distribution for the draft genome of *Arthrobacter* sp. ES1 generated from RAST.

This data set includes raw and assembled DNA sequences that have been quality-assessed, as well as annotated versions of the genomes of *Arthrobacter* sp. ES1. The resulting paired-end sequencing reads were designated as ES1_R1.fastq and ES1_R2.fastq. Herein, the raw and clean-sequencing reads, statistics for the assembly, the genome's quality, and its annotation are reported. A total of 3,248,048 raw reads were generated resulting in 490,455,248 bases (Table 1). The sequencing reads were then pre-processed to remove low-quality, contaminant, and short reads, a total of 72.92% of clean reads were recovered. The genome size of strain ES1 is 3,964,927 bp at 77 × sequence coverage with a GC content of 65.73%. The strain ES1 draft genome consists of 170 contigs, with the longest contig having 356,645 bases, the N50 having 66,568 bases, and the N90 having 15,117 bases. *De novo* assembly produced 111 small contigs (<10,000 bp) and 59 large contigs (>10,000 bp) (Table 2). The quality of the draft genome of strain ES1 was examined using Benchmarking Universal Single-Copy Ortholog (BUSCO) tested with actinobacteria_odb9 lineage, resulting in 97.8% of complete BUSCOs (Fig. 1). The genome annotation was performed using Rapid Annotation using Subsystem Technology (RAST) server. The output shows that there are 3,904 coding sequences and 51 RNAs in strain ES1, and 25% of coding sequences were classified into 285 subsystems (Fig. 2).

**Table 1:** Pre-processed sequencing reads statistics of forward (ES1_R1.fastq) and reverse (ES1_R2.fastq) reads.

Description	Forward	Reverse	Total
Total Raw Reads	1,624,024	1,624,024	3,248,048
Total Raw Reads Bases	245,227,624	245,227,624	490,455,248
Total Clean Reads	1,357,978	1,010,480	2,368,458
Total Clean Reads Bases	186,557,107	117,766,675	304,323,783
Clean Reads (%)	83.62	62.22	72.92
GC Content Clean Reads (%)	64	64

**Table 2:** Assembly statistics for the draft genome of *Arthrobacter* sp. ES1

Feature	Value	Percentage (%)
Genome size	3,964,927	
Numbers of contigs	170	
N50	66,568	1.679
N90	15,117	0.381
L50	18	
GC content (%)	2,604,737	65.73
Longest contig	356,645	8.995
Shortest contig	201	0.005
Number of contigs < 8k bases	90	52.941
Number of contigs > 8k bases	21	12.353
Number of contigs > 16k bases	54	31.765
Number of contigs > 100k bases	4	2.353
Number of contigs > 200k bases	1	0.588
Mean contig size	23,323.1	0.588
Median contig size	7,247	0.182

## 3 Experimental Design, Materials, and Methods

### 3.1 Genome DNA extraction and sequencing

*Arthrobacter* sp. ES1 was grown in nutrient broth (NB) medium at 20°C for 3 days and used for genomic DNA extraction. Genomic DNA was extracted by using DNeasy Blood and Tissue kit (Qiagen, Inc, USA) according to the manufacturer's instructions. The Nextera® XT DNA sample preparation kit was used to construct a genomic library. A whole genome shot-gun sequencing was performed by using a 300 cycles Miseq® Reagent Kit v2 on an Illumina MiSeq sequencer to generate 150 bp paired-end reads.

### 3.2 Reads Pre-Processing, Genome Assembly, Quality Assessment, and Annotation

The raw reads were pre-processed with the SolexaQA tool to remove low-quality bases (Qphred < 20) and short reads (minimum length=50) [1]. Reads were filtered using bowtie2 to remove phiX reads [2]. FastQC was used to ensure that the generated clean reads were of high quality (https://www.bioinformatics.babraham.ac.uk/projects/fastqc/) [8]. *De novo* assembly and scaffolding were performed by using Velvet v1.2.10 [3]. The quality of the draft genome was assessed by using Benchmarking Universal Single-Copy Ortholog (BUSCO) [4]. The draft genome was annotated by using Rapid Annotation using Subsystem Technology (RAST) software [5].

## Ethics Statement

This work neither involves human subjects nor animal subjects. The authors declare that this manuscript is original work and has not been published elsewhere.

## CRediT authorship contribution statement

**Chui Peng Teoh:** Conceptualization, Methodology, Data curation, Writing – original draft, Writing – review & editing. **Nur Athirah Yusof:** Conceptualization, Methodology. **Cahyo Budiman:** Conceptualization, Methodology. **Yoke Kqueen Cheah:** Conceptualization, Methodology. **Clemente Michael Vui Ling Wong:** Conceptualization, Methodology, Supervision, Writing – review & editing.

## Declaration of Competing Interest

The authors declare that they have no known competing financial interests or personal relationships that could have appeared to influence the work reported in this paper.

The authors declare the following financial interests/personal relationships which may be considered as potential competing interests:

## Data Availability

WGS of Arthrobacter sp. ES1: Pure culture (Original data) (NCBI SRA database).
